# Metacognitive Control of Categorial Neurobehavioral Decision Systems

**DOI:** 10.3389/fpsyg.2016.00170

**Published:** 2016-02-19

**Authors:** Gordon R. Foxall

**Affiliations:** Cardiff UniversityCardiff, UK

**Keywords:** competing neuro-behavioral decision systems, CNDS model, dual- and triple-process models, metacognitive control, temporal discounting, picoeconomics, bundling, categorical system

## Abstract

The competing neuro-behavioral decision systems (CNDS) model proposes that the degree to which an individual discounts the future is a function of the relative hyperactivity of an impulsive system based on the limbic and paralimbic brain regions and the relative hypoactivity of an executive system based in prefrontal cortex (PFC). The model depicts the relationship between these categorial systems in terms of the antipodal neurophysiological, behavioral, and decision (cognitive) functions that engender normal and addictive responding. However, a case may be made for construing several components of the impulsive and executive systems depicted in the model as categories (elements) of additional systems that are concerned with the metacognitive control of behavior. Hence, this paper proposes a category-based structure for understanding the effects on behavior of CNDS, which includes not only the impulsive and executive systems of the basic model but a superordinate level of reflective or rational decision-making. Following recent developments in the modeling of cognitive control which contrasts Type 1 (rapid, autonomous, parallel) processing with Type 2 (slower, computationally demanding, sequential) processing, the proposed model incorporates an arena in which the potentially conflicting imperatives of impulsive and executive systems are examined and from which a more appropriate behavioral response than impulsive choice emerges. This configuration suggests a forum in which the interaction of picoeconomic interests, which provide a cognitive dimension for CNDS, can be conceptualized. This proposition is examined in light of the resolution of conflict by means of bundling.

## Introduction

“…akrasia in rational beings is as common as wine in France”

(Searle, 2001, p. 10)

As I scan my daily newspaper over breakfast, I note the television programs scheduled for the evening. It is easy at so early an hour to vow that I will under no circumstances allow myself to watch what is on offer. Tidying my sock drawer or deadheading the roses seems a more valuable use of my time, and serious reading or writing infinitely preferable. Comes the evening, however, the opportunity to relax and be passively entertained wins out. Am I speaking here of “myself” as one person whose preferences are reversible simply with the passage of time, or of two separate categories of agents warring to get the upper hand? If the latter, how are these categories related and how do they influence each another? Perhaps there is some superordinate level of decision-making that arbitrates between them; or perhaps they reflect no more than differing histories of operant reinforcement.

The problem of preferences that change with time lies at the heart of many comparatively trivial daily decisions. What seems perfectly reasonable when we begin becomes absurd simply because other options, ultimately less valuable than the initial longer-term objective, have become immediately accessible (Rachlin, 2000a). Apparently for that reason alone, these choices that may be categorically classified as temporarily short-term assume an irresistible level of attractiveness: the result may be excessive consumption leading to obesity or procrastination leading to failure to achieve (Ainslie, 2010). The problem, akrasia or weakness of will, occurs also in the more serious contexts of substance abuse and problem gambling, even when the individuals concerned know from experience the deleterious outcomes of their behavior and have the “best intentions” of changing it. Once again, the questions of the apparently “divided self” or “multiple selves” arise (Elster, 1987; Ainslie, 2001; Ross et al., 2008). It is interesting that Searle (2001) speaks of akrasia as a common characteristic of *rational* beings: in what sense are we to understand the rationality that underlies such self-defeating behavior?

The initially self-controlled and subsequently impulsive behaviors involved in preference reversal can be traced to neurophysiological and cognitive bases of competing decision systems. Jentsch and Taylor (1999) propose that drug seeking stems from amygdala-based reward processing that intensifies the incentive value of potentially addictive substances, accompanied by the weakened capacity of frontal cortical processes to impede such behavior. Bechara (2005) similarly argues that the extent of an individual’s willpower to resist drugs depends on the relationship between an impulsive system based on the amygdala which indicates the immediate outcomes of behavior and a reflective system, based on ventromedial prefrontal cortex (vmPFC) which indicates delayed outcomes (Bickel and Yi, 2010). This relationship has been most comprehensively described, however, in the competing neuro-behavioral decision systems (CNDS) model which hypothesizes that two competing neural systems, respectively, exert excitatory or inhibitory control over potentially addictive behavior (Bickel et al., 2013).

The CNDS model proposes that imbalance between an individual’s “impulsive” and “executive” categorical systems influences his/her rate of temporal discounting (see **Box 1**).^1^ Hyperactivity of the impulsive system, based on limbic and paralimbic brain regions, coupled with hypoactivity of the executive system, based in the prefrontal cortex (PFC), results in a tendency to discount the future steeply and to engage in addictive behavior (Bickel and Yi, 2008). A major premise of the CNDS model is, therefore, that the impulsive and executive systems must be in some respects antipodal categories and yet contribute in a complementary manner to the determination of the individual’s temporal discounting behavior and valuation of currently and potentially available reinforcers. These have been concerns of the CNDS model’s authors who also emphasize the role of metacognition (i.e., “cognition about cognition” or “thought about thought”) in the regulation of inter-system connectivity (Jarmolowicz et al., 2013). In attempting to clarify further the factors responsible for the achievement of relative balance between the impulsive and executive systems, this paper explores further the antipodality of the model’s categorical component decision systems and, in particular, the nature and role of metacognition in their relationships.

**Box 1. Temporal discounting and preference reversal.**

A reward that is to be received at some time in the future – say, $100 in a year’s rime – does not seem right now to be worth waiting that long for unless there is some extra bonus attached to it. If someone owes me this amount and offers to let me have in 12 months, I am inclined to say that I will require, say, $110 at that time. Reward for which one has to wait are devalued or discounted. We say that temporal discounting is concerned with the *current subjective value* of a reward that will be received in the future, i.e., the value of that future reward rated in the present moment. Financial professionals discount exponentially, i.e., at a constant rate regardless of the time elapsed. Their behavior can be expressed as *Vi* = *A_*i*_e*^–^*^*kDi*^* where *Vi* is the present value of a delayed reward, *Ai* the amount of delayed reward, *k* a constant proportional to the degree of temporal discounting, *Di* the delay of the reward, and *e* the base of natural logarithms. Because this behavior is based on a constant rate of discounting, a larger, later reward (the LLR, available at *t_2_*) *always* has a value greater than that of a smaller reward available sooner (the SSR, available at *t_1_*). This is shown in the first segment **(A)** of the figure, where the two lines, representing the relative values of the reward, never cross.

Often, however, human behavior is marked by a style of discounting in which the value of a reward changes radically as the time remaining before it becomes available is reduced. While the LLR is preferred at *t_0_*, indicated by the initially higher line in segment **(B)** of the figure, just prior to *t_1_, when* the SSR will becomes available, its value markedly increases, the curves cross, and the individual opts for the poorer reward. This form of temporal discounting and the preference reversal it involves is described by a hyperbolic function: *V_*d*_ = A/*(*1 + kD*) in which *V_*d*_* is the discounted value of a reward of a particular magnitude or amount, *A*, received after a delay, *D* (Mazur, 1987; Madden and Bickel, 2010). Rate of discounting varies with the amount of delay (Ainslie, 1992, 2001; Rachlin, 2000a; Rick and Loewenstein, 2008).

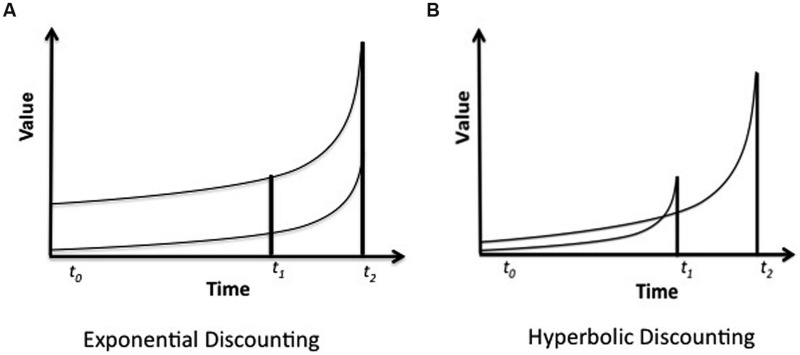

The CNDS model has two important implications for the resolution of the question of multiple selves. First, by incorporating *cognitive* or decision-making contributors to the extent of an individual’s temporal discounting tendency, it links to the capacity to regulate behavior through goal setting and maintenance, social cognition (understanding why others behave as they do), and insight (taking one’s own imperfections into account in judging behavioral outcomes). Second, the model’s incorporation of operant behavioral economics and neuroeconomics (Bickel et al., 2007, 2011, 2012a) facilitates its integration with the economic reasoning which underlies another significant contribution to the explanation of multiple selves and their interaction, namely *picoeconomics* (Ainslie, 1992, 2001; Ross, 2012; Foxall, 2014a,b).

**FIGURE 1:**
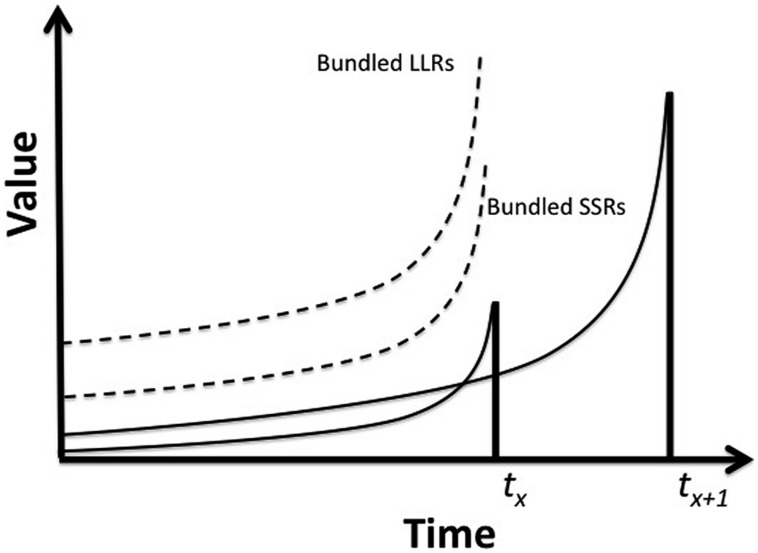
**The Principle of Bundling.** The solid lines represent any of the individual members of the stream of paired SSR/LLR choices that will be available to the individual over time. (Hence, *t_x_* is the time of any occurrence of an SSR which is paired with an LLR that occurs at *t*_*x*+1_, and we are assuming a sequence of such SSR/LLR pairings over time). The dashed lines represent the individual’s imagined aggregation of these reward if they were all brought forward to a point just prior to the appearance of the first SSR. In this case, LLR will always exceed SSR and a decision to select it exclusively on subsequent occasions can be more easily made.

Ainslie (1992) speaks of the problem of akrasia by reference to separate *interests* that are in conflict: one concerned with our gaining long term benefit such as engaging in productive work, the other with short-term pleasures like undemanding amusement. One’s experience as the locus of this clash of interests is often marked by a sturdy resolve to undertake the more rewarding activity, followed by a lapse into the other just as it becomes available, followed by regret, further resolution and perhaps inevitable relapse. This cycle is characteristic of addiction but it also marks many everyday switches of preference involved in less extreme behavior. What is so preferable when we make our plans is edged out by an alternative that is initially unthinkable but of immense value as it arrives in sight. Even though we know full well that the activity which we were determined to undertake when we set out will bring greater benefit, the fact that it is delayed while the less beneficial can be obtained immediately raises the value of the latter sharply till it exceeds the current worth of the other (see **Box 1**). An intriguing facet of Ainslie’s approach is the possibility that, by “bundling” together (**Figure 1**) the combined benefits of a series of later-appearing reward and comparing these *in toto* with the immediate benefit of a current less valuable choice, it is possible to overcome the temptation to make a sub-optimal decision (i.e., to exercise “willpower” or “self-control”). Hence, picoeconomics has implications for the role of cognition and metacognition in relationships between neuro-behavioral decision systems and the place of agency in understanding their interaction.

Some of these implications are taken up by Ross (2009) who defines the situation in economic terms by reference to two reward available at different times such that *a* is, for example, taking a short vacation starting in a week [*t*1], and *b* is, for instance, starting a 2-years course of study for a higher degree, [*t*2]. Looking well into the future, the person’s utility function indicates that *b* is preferable to *a*. At this point, the person discounts the future rather gently. However, as the time for the vacation to occur becomes closer, the person’s utility function indicates a preference for *a* over *b.* Ross (2012) models the various picoeconomic interests in two ways depending on whether these interests are conceived as acting synchronously or diachronically. In the first case, they may be seen as subagents that have either conflicting utility functions or divergent time preferences. Agents with conflicting utility functions may be modeled in terms of a Nash equilibrium game among these agents. Modeling the behavior of subagents whose time preferences diverge adverts to the sub-personal level of neurophysiology in which a hyperbolic time preference emerges from “competition between steeply exponentially discounting ‘limbic’ regions and more patient (less steeply exponentially discounting) ‘cognitive’ regions” (Ross, 2012, p. 720). Like the CNDS model, this picoeconomic portrayal depends heavily on the findings a key experiment in neuroeconomics based on fMRI scans of humans choosing between SSR and LLR (McClure et al., 2004). In the process of scrutinizing immediate reward, participants activated brain regions that involve emotion, namely medial orbitofrontal cortex, medial prefrontal cortex/pregenual cingulate cortex, and ventral striatum. However, while examining longer-term payoffs, they activated areas of the lateral PFC (implicated in higher cognitive functioning), and part of the parietal cortex related to quantitative reasoning. In his modeling of picoeconomic conflict in terms of diachronically appearing multiple selves, Ross (2012) speculates briefly about the cognitive demands of such a portrayal: each subagent is portrayed as temporarily in control of the person’s behavior, with its own utility function and incomplete knowledge of the other, though its utility is constrained by the investments made by earlier-appearing agent(s).

In seeking to clarify the issue of multiple selves, this paper draws on recent investigations of antipodality between the categorial components of the impulsive and executive systems (Bickel et al., 2012b). This work is invaluable for identifying the elements of a theory of behavior that would account for both normal and excessive (addictive) consumption of substances such as alcohol and other drugs and activities such as gambling. Importantly, it demonstrates which elements of the impulsive system are antipodal to elements of the executive system (and can, therefore, be properly considered categorial components of these antithetical tendencies), as well as those which play a broader role in the execution of appropriate behaviors. Prominent among the latter are what the CNDS model identifies as metacognition and the goal-directed regulation of behavior (Jarmolowicz et al., 2013).

The paper builds on the results of this work to propose a model of cognitive functioning in addiction that places the impulsive and executive systems in a framework consistent with recent developments in multi-process theories of cognition (Stanovich and West, 2000; Stanovich, 2009, 2011). It thereby incorporates a broader domain of theory on the cognitive control of behavior which acknowledges a long-standing division of thinking into that which is rapid and intuitive as opposed to that which is slow and deliberative (Evans and Stanovich, 2013). This dichotomy of categories is similar to that which marks the distinction between impulsive and executive systems and is consistent with the account of behavioral control that the examination of the CNDS model in terms of antipodality reveals. The advantage of such a framework is that it allows for a forum within which the competing demands of the impulsive and executive systems interact so that conflict is resolved and behavior that generates more acceptable long-term consequences is selected over short-term expediency.

The central focus is, therefore, on the structure of the CNDS model and, in particular, its incorporation of metacognitive control of behavior. In the next sections, the CNDS model is described in greater detail and the implications of antipodality for the construal of decision systems is discussed. The questions of how metacognition is depicted in the model and a potential tripartite model are the foci of the following sections. Finally, the implications of the analysis for understanding the multiplicity of selves involved in the decision process are discussed by critically examining the emergent framework in terms of picoeconomic bundling behavior.

## The Competing Neuro-Behavioral Decision Systems Model

### The Neurophysiological Dimension

At the neurophysiological level, the CNDS hypothesis (Bickel et al., 2012a, 2013) assumes that normal and addictive behaviors reflect the balance between the relative hyperactivity of the limbic and paralimbic systems that are differentially implicated in emotional responding and the relative hypoactivity of prefrontal cortical areas that are differentially implicated in judgment, planning, and other cognitive activities. Hence, the degree of addictiveness exhibited in behavior reflects the balance of activity in brain regions, the first of which, the *impulsive system*, based on the amygdala and ventral striatum, involves the distribution of dopamine during reinforcement learning, while the second, the *executive system*, residing in the PFC, is implicated in the evaluation of reward and their outcomes. The competing systems that comprise the model are more broadly based than these neurophysiological regions, embracing in addition behavioral and cognitive components which justifies their being called neuro-behavioral decision systems. (Bechara, 2005, nominates these the impulsive system and the reflective system, respectively; see also Verdejo-Garcia and Bechara, 2009).

#### The Impulsive System

The impulsive system incorporates the amygdala and ventral striatum, a midbrain region concerned with the valence of immediate results of action, and is liable to become hyperactive as a result of “exaggerated processing of the incentive value of substance-related cues” (Bechara, 2005, p. 1459). Drug-induced behaviors correlate with enhanced response in this region when the amygdala displays increased sensitization to reward (London et al., 2000; Bickel and Yi, 2008). The receipt of positive reinforcers of all varieties causes the release of dopamine in the nucleus accumbens. This is true of both utilitarian reinforcers such as drugs of abuse, and the receipt of informational reinforcers such as social reward or self-esteem (Foxall, 2011). It is also the case for of the receipt of money which has both utilitarian and informational aspects. In the case of a drug of abuse, such brain reward is acute. The effect of the drug in inculcating LTP at specific synapses is recorded in the hippocampus as the result of experience (memory).^2^ In the amygdala is involved in the creation of a learned (conditioned) response to the stimuli that accompany the use of the drug. These accompanying stimuli might take the form of informational (social) reinforcers and discriminative stimuli. (For discussion of these points, see, inter alia, McGaugh, 2004; Phelps, 2006; Gruber and McDonald, 2012.)

The resulting focus of research has been on the mesolimbic dopaminergic system and other brain regions such as the amygdala and ventral striatum involved in emotional responses. But there is recent evidence that the insula is important because of its relation to conscious craving for drugs (Naqvi and Bechara, 2008). This role has been revealed by correlation-based fMRI studies which show the increased activity of the insula during self-reported urges to ingest drugs. Such activity is related to the emergence of the secondary reinforcers which tie drug use to specific behavioral and contextual factors and to the cognitive drivers of drug use. “Over time, as addiction increases, stimuli within the environment that are associated with drug use become powerful incentives, initiating both automatic (i.e., implicit) motivational processes that drive ongoing drug use and relapse in addiction to conscious (i.e., explicit) feelings of urge to take drugs” (Naqvi and Bechara, 2008, p. 61; see also Naqvi et al., 2006). The ritualistic practices involved in the preparation of drugs, associated with specific places, apparatus, packages, lighters, and so on, thus become sources of the pleasure that reinforces not only those activities but the consummatory acts of drug ingestion. These processes, which elicit specific memories of encounters with the contexts and the drugs, are also responsible for differences in the subjective experience of urges for various drugs be they cigarettes, cocaine or gambling. By ensuring that the individual keeps particular goals “in mind,” the insula is also involved in (thwarting) the executive functions that might overcome drug urges (cf. Tiffany, 1999). The learning process includes the development of neural plasticity through DA-priming with respect to the impending chain of appetitive events; Naqvi and Bechara (2008) propose that this DA-dependency invokes activity in the insula and associated regions such as the VMPFC and amygdala. The plasticity involves the establishment of representations of the interoceptive outcomes of using drugs and thus engender relapse even after long periods of non-use.

#### The Executive System

The executive system, which includes the PFC is normally associated with the executive functions of planning and foresight (Barkley, 1997, 2012), and is hypothesized to become hypoactive in the event of addiction. In the absence of its moderating function, effects of the hyperactive dopaminergic reward pathway are exacerbated, leading to an imbalance which is implicated in the enactment of dysfunctional behavior. The behavioral concomitant of these neurophysiological processes is observable in the rate at which individuals discount the value of future reward in favor of more-immediately appearing reinforcers (Bickel and Yi, 2008). In the context of addiction, the CNDS hypothesis posits that drug seeking results from “amplified incentive value bestowed on drugs and drug-related cues (via reward processing by the amygdala) and impaired ability to inhibit behavior (due to frontal cortical dysfunction)” (Bickel and Yi, 2010, p. 2).

Analysis of the neurobiological pathway proposed to account for the acquisition by the PFC of the capacity to control the higher-level cognitive functions involved in the regulation of behavior in the face of environmental programming reveals a two-stage process (Miller and Wallis, 2009). The first stage is the impingement of signals generated via reinforcement learning on the PFC circuitry: reinforced operant behavior is accompanied by the production of signals that associate PFC functioning with aspects of the stimulus field (the setting in which the behavior takes place), the nature of the behavioral response enacted, and the reinforcing and punishing consequences that are its outcomes. Repeated responding in these circumstances is capable of generating strong PFC representations of the contingencies of reinforcement that maintain such behavior. The second stage in the argument is to account for these signals and the actions of dopaminergic neurons of the midbrain. In the course of learning through the repeated performance of behavioral responses, reinforcers initially activate the dopaminergic neurons themselves, but subsequently the stimuli that predict the reinforcers, rather than the reinforcers themselves, come to activate the dopaminergic neurons. Should an expected reward not appear, the rate of firing of the dopaminergic neurons is reduced. The discrepancy between the expectation of reinforcement and its non-appearance, coded by the dopaminergic neurons’ activity is known as the *reward prediction error* and is instrumental in the organism’s subsequent ability to direct its actions more effectively toward the achievement of reinforcement (Miller and Wallis, 2009, p. 103–104; see also Foxall, 2014b).

The fundamental assumptions that reinforcement is coded by dopaminergic neurons (Schultz, 1992; Robbins and Everitt, 2002) and that RPEs are also reflected in the firing rates of dopaminergic neurons (Schultz et al., 1997) ground the relationship between neoclassical micro-economics and neuroscience on which neuroeconomics rests (Glimcher, 2011). For present purposes, they serve to integrate operant psychology with these disciplines by promoting a causal connection among reinforcement, neuronal activity, and behavior (Schultz and Dickinson, 2000; Schultz, 2010).

### The Behavioral Dimension

The operations of these systems combine to generate behavior that reflects the individual’s valuation of future events, his/her degree of “temporal discounting.” Hyperbolic temporal discounting is the procedure in which the later-occurring of two reward is diminished in an individual’s subjective estimation even though it is the larger, with the result that the more immediate reward is selected in preference despite its being by definition the smaller of the two. This “impulsive” behavior is described by the hyperbolic discounting function

$$V_{d}=A/1+k\,D\,\;\;\;\;\;\;\;\;\;\;\;\;\;\;\;\;\;\;\;\;\;\;\left(1\right)$$

where *V_d_* is the discounted value of a reward of a particular magnitude or amount, *A*, received after a delay, *D* (Mazur, 1987; Madden and Bickel, 2010). The *k* parameter indicates the extent to which the value of the LLR diminishes compared to that of the SSR over time (Stein and Madden, 2013). The major behavioral characteristic of choice described hyperbolically is that the individual is likely to reverse preferences as time advances, an observation which is highly relevant to the extreme drug-use and gambling already mentioned, the making of resolutions to change, and the yielding to temptation that may follow. Behavior that discounts the future is of central importance to the CNDS model insofar as temporal discounting is an index of the extent to which behavior is under the control of the tendency toward disinhibited impulsivity (the selection of an SSR rather than an LLR) as opposed to the inhibiting influence of the executive functions which results in the choice of LLR over SSR (“self-controlled” behavior) (Bickel and Marsch, 2000; Bickel and Yi, 2008; Barkley, 2012).

It is reasonable to inquire how the valuation an individual attaches to the outcomes of his/her future behavior should be understood. The CNDS model argues that the neurophysiological tendencies of the impulsive and executive systems eventuate in an individual’s degree of temporal discounting behavior which is explicable in operant terms that translate readily into economic considerations (Bickel et al., 2007, 2011). Bickel et al. (2012a) argue that addiction can be conceptualized as an outcome of “reinforcer pathologies” that can be analyzed in terms of behavioral economics, specifically the inelasticity of demand (manifesting in a willingness to pay an extraordinarily high price for a drug reward) and extremely steep discounting of the future (manifesting as over-valuation of an immediately available reward). These elements which reveal an excessive valuation of one reinforcer in comparison with other available reward and impulsivity, respectively, are consistent with the pattern of behavior found in addicts who may accordingly be defined as “people for whom the transient benefits of the addictive behavior persistently outweigh the significant short- and long-term costs of these choices” (Bickel et al., 2012a, p. 334–335). The portrayal of these benefits in terms of positively- and negatively reinforced behaviors is confirmed by the neurophysiological analysis of addiction which depicts addicts’ initial drug administration as determined by the pleasures this confers and their later drug use as a means of avoiding or escaping from deleterious consequences such as withdrawal symptoms (Koob, 2013). Some aversive consequences cannot be avoided by further drug administration, however; the social isolation and damage to health that often result from persistent addiction are examples of the punishing outcomes of such behavior (Rachlin, 2000b; Foxall and Sigurdsson, 2011).

### The Need for a Cognitive Dimension

There are several reasons for thinking more formally about the place and function of a cognitive dimension within neurophysiologically based models of decision-making. The principal reason in the current context stems from the fact that the “valuation” involved in temporal discounting is a mental construct which requires explanation in terms of cognitive representation and evaluation. This, in turn, raises the concern that the present dual process structure of the model may be inadequate to the task of accounting for the metacognitive processes involved in the exercise of self-control. In the discussion that follows, the distinction between the sub-personal level of exposition, that concerning brains and neuronal activity, and the personal level of exposition, that which involves intentionality (e.g., desires, beliefs, emotions, and perceptions) and behavior (Dennett, 1969), is not only of primary importance but inviolate (Foxall, 2007).

## Antipodality

### Bases of Antipodality

The CNDS model is an example of a dual process theory, i.e., one that builds on a substantial volume of social scientific argument that human cognition is characterized by two categorial styles of processing (Frankish and Evans, 2009). Type 1 processing is *autonomous:* its execution is *rapid* and *mandatory*, economizes on central processing capacity and higher-level control systems, and employs *parallel* processing so that it avoids interfering with other cognitive operations. These characteristics illustrate the *computational ease* that makes Type 1 processing the default processing mode: unless it its overridden, it will automatically generate a category of responses to environmental conditions. Type 1 processing includes the regulation of behavior by the emotions, encapsulated modules that solve adaptive problems, implicit learning processes and the automatic firing of overlearned associations. By comparison with the Type 1 thinking that characterizes the *autonomous mind* (Stanovich, 2009), the second category of processing, type 2, is *slow* and makes *heavy computational demands*. It requires attention, which is costly, and is involved in conscious problem solving, eventuating in behavior that is directed toward achieving long-range consequences.

**FIGURE 2:**
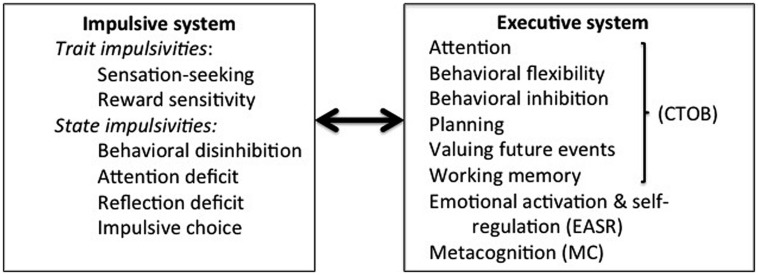
**Competing Neuro-behavioral Decision Systems (CNDS).** This depiction shows the interaction of the impulsive and executive systems which are delineated in terms of the components as identified by Bickel et al. (2012b).

The distinction between the impulsive and the executive systems, and that between their respective styles of processing, suggests at least two categorial bases of evaluation and judgment that have opposing tendencies toward behavioral outcomes. This implies that at the systems can be construed as antithetical in important respects that can be related to their interaction to produce particular observed behavior patterns. These antipodal tendencies of the impulsive and executive systems ought ideally to indicate why behavioral imbalance would result from the hyperactivity of one system simultaneously with the hypoactivity of the other, a possibility which Bickel et al. (2012b) have investigated. These authors propose eight executive functions relevant to the CNDS model: Attention, Inhibitory control, Valuing future events, Cognitive behavioral flexibility, Working memory, Planning, Emotional activation and self-regulation, and Metacognitive processes. The first four are categorized as concerned with the *cross-temporal organization of behavior* (CTOB). *Emotional activation and self-regulation* (EASR), comprising two elements: Processing of emotional information and Initiating and maintaining goal-related responding, and *metacognition* (MC) comprising two more: Social cognition (consisting in theory of mind, empathy) and Insight (or self-awareness). In addition, they propose as elements in the impulsive system, two trait impulsivities: Sensation seeking and Sensitivity to reward, and four state impulsivities: Behavioral disinhibition, Attention deficit impulsivity, Reflection impulsivity, and Impulsive choice (preference for SSR over LLR). The impulsive and executive systems, delineated in terms of the components identified by Bickel et al. (2012b), are shown as interacting systems in **Figure 2.**

Bickel et al. (2012b) assess antipodality by reference to four criteria: definition, measurement, overlapping of clinical populations, and commonality of neural substrates of the elements of impulsivity and executive function that comprise the CNDS model. It emerges from their analysis that the four state impulsivities are definitionally antipodal to four of the executive functions. Attentional deficit impulsivity and attention are clearly opposites, while the definitions of behavioral inhibition and behavioral disinhibition contain common characteristics that set them apart. In addition, reflection impulsivity tends toward the opposite of planning. Finally, the selection of SSR over LLR is antipodal to the capacity to value future outcomes. Note that the four executive functions identified as having antipodal impulsivities all belong to the CTOB grouping.

The analysis of antipodality also reveals a categorically distinct though coterminous measures of impulsive system and executive system items in the case of attention-deficit impulsivity and attention, and for behavioral inhibition and behavioral disinhibition. Reflective impulsivity and planning are less similar in their measurement. Finally, the delay discounting methods employed to measure impulsivity have recently come to be used in the measurement of executive functions. Note once more that these results establish CTOB as the seat of executive function which is the antipode to impulsivity.

The third source of evidence is the overlap of clinical populations whose members suffer from addiction and who show either hypoactive executive function or hyperactive impulsivity. Some substance users/abusers for instance demonstrate response inhibition deficits and excesses in behavioral disinhinbition. When the substance is alcohol, this tends to be accompanied by lack of attention on the one hand and exaggerated attention deficit impulsivity on the other. Deficits in planning and high levels of reflection impulsivity are found in users of amphetamines, cigarettes, and opiates. Finally, addicts to alcohol, cigarettes, cocaine, and heroin display steeper discounting of delayed reward more than controls do. Executive function deficits are also closely related to drug addiction.

Finally, in terms of the overlap in neural substrates of brain regions implicated variously in the functioning of the impulsive and executive systems, it is noteworthy that the insula and parts of the PFC are implicated in both behavioral disinhibition and behavioral inhibition. Moreover, since choice impulsivity and the valuation of future events are measured by means of delay discounting assignments they must recruit the same brain areas; they also cite the strongly emerging evidence that the limbic and paralimbic areas are implicated in immediate choice whilst parts of the PFC are implicated in the selection of delayed reward (and therefore with the valuation of future events). Again, it is noteworthy that all of these executive functions belong to the CTOB category. There is, however, little evidence of any overlap between the neural substrates of reflection impulsivity and planning other than the observation that individuals with lesions to the frontal cortices exhibit high reflection impulsivity which supports the view that DLPF and DMPFC are concerned with planning. There is also a paucity of evidence for any neural overlap for attention and attention deficit impulsivity. Nor is impulsivity antipodally related to working memory, EASR or MC even though impediments to these are found variously in addiction. Overall we may conclude that CTOB is antipodally related to the state impulsivities by evidence that they implicate similar neural substrates but that there is little evidence that the other elements employed in the categorization of executive functions shown in **Figure 2** are similarly related to impulsivity.

This does not constitute an original critique of the CNBDS model; indeed, the points made are all acknowledged by Bickel et al. (2012b). These authors specifically note that working memory answers no antipodal aspect of impulsivity and they draw attention to the lack of antipodal relationship between EASR and MC on one hand and impulsivity on the other. Such a relationship would be expected if EASR and MC belonged to executive functions. However, this examination of the findings suggests an alternative depiction of how the impulsive system and executive system are related.

### Locating Metacognition

Since the evidence for antipodality locates the competing classes of activity of the impulsive and executive systems firmly within neurophysiological bases, the task remains of placing the decision-making or cognitive elements of CNDS, currently located in the executive System (**Figure 2**), for which no evidence of corresponding and antipodal functions within the impulsive system has been adduced. There are in fact good reasons for separating metacognition (MC) and emotional activation and goal regulation (EASR), in which these cognitive or decision-making functions inhere, from the neurophysiological dimensions which are demonstrably antipodal. Because both MC and EASR involve thought or feeling about thought or feeling, I shall refer to them collectively as *metacognition*, though there may not exclusively fulfill this role in explaining human behavior. The justification for treating these cognitive variables as involved in the explanation of behavior is as follows.

**FIGURE 3:**
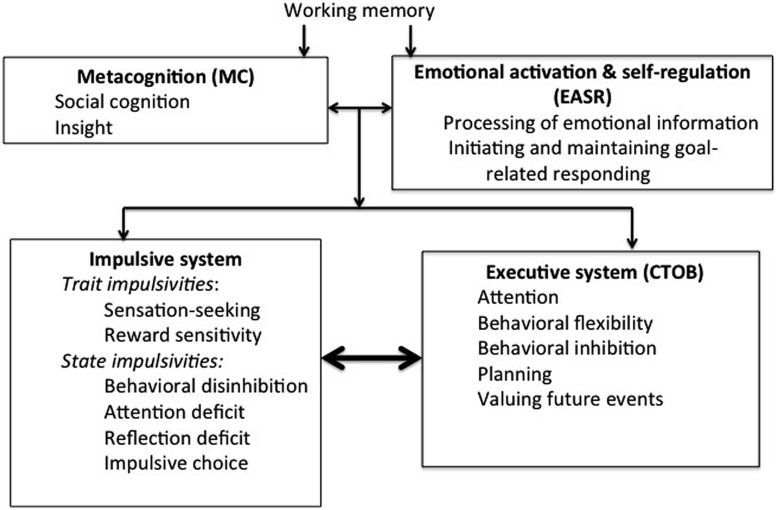
**Separation of Metacognition (MC) and Emotional Activation and Self-Regulation (EASR) from the Executive System.** This depiction shows the MC and EASR components separately from the executive system since they have no apparent antipodal correspondents in the impulsive system. This is suggestive of their exerting a superordinate influence over the impulsive and executive systems and their interactions. It is therefore indicative of the necessity of developing a tripartite model of the cognitive control of CNDS.

If the CNDS model were conceived solely in terms of the neurophysiologically defined impulsive and executive systems that have been shown to be antipodal, then the individual’s behavior manifested in a degree of temporal discounting peculiar to him/her would be the outcome of a sub-personal battle between opposing biological forces. Behavior would be starkly determined by innate neurophysiological capacities resulting from phylogenetic evolution, modified by a learning history that results in neural plasticity formed in a process of Hebbian or similar learning (Rolls, 2008). Behavior would be no more than contingency-shaped, determined in its totality by contingencies of natural selection and operant conditioning. However, this would be to ignore the rule-governance of behavior, the possibility of an influence of reflective thought on responding. By including MC and EASR in their model, Bickel et al. (2012b) take this into account. Their inability to find or suggest functions of the impulsive system that are antipodal to these cognitive functions, which in any case repose uneasily among the other elements of the executive system depicted in **Figure 2**, argues for their separate consideration. The resulting re-conceptualization is shown in **Figure 3** in which MC and EASR are shown separately from the executive system which contains only those elements that are demonstrably antipodal to elements of the impulsive system. The impulsive system retains *pro tem* the trait impulsivities, sensation-seeking and reward sensitivity, that have no demonstrable correspondents in the executive system. **Figure 3** indicates also the reliance of MC and EASR on working memory.

The empirical outcome of the search for antipodality between impulsive and executive systems represented by Bickel et al.’s (2012b) research suggests the outcome shown in **Figure 3.** But there is a theoretical imperative for the proposal that metacognition occupy a superordinate position to the competing impulsive and executive systems. If the conflict of these systems is to be resolved by means of “cognition about cognition” or “thinking about thinking,” it follows that such metacognitive activity must take place in a forum separate from the categorial systems themselves: how else could such activity decide between the interests these systems underpin? As a judge always sits apart from and acts independently of the advocates of plaintiff and defense, the realm of mediation, intercession, and arbitration in decision-making cannot be incorporated within either the short-range interest that tends toward immediate gratification or the long-range interest that seeks a wider echelon of optimization.

## A tri-Process Model

### Structure of the tri-Process Model

Recent theoretical development in multi-process theory suggest that metacognitive processes such as MC and EASR are most appropriately positioned as superordinate to the interactions of impulsive and executive systems as well as other systems that influence their interrelationship (Stanovich, 2009). Since no antipodal relationship between MC and EASR on one hand and components of the impulsive system on the other suggests that this possibility at least be considered as a means of understanding more fully the import of the CNDS model.

**FIGURE 4:**
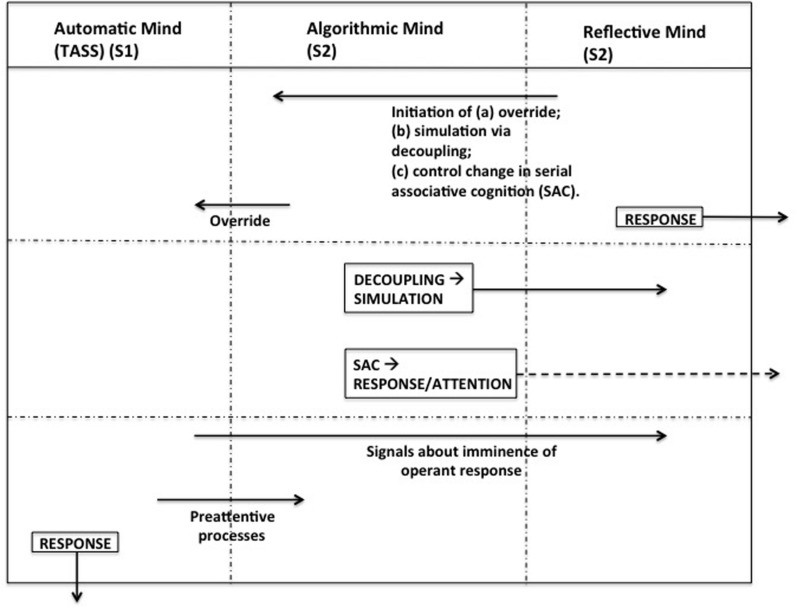
**Essential relationships in Stanovich’s tripartite theory**.

The similarity of the Type 1/Type 2 dichotomy to that of the Impulsive system/Executive system distinction is readily apparent. But any conclusions about the structure of the CNDS model in terms of these different styles of processing should take account of Stanovich’s proposal for a tri-process theory (**Figure 4**). In proposing such a structure, Stanovich (2009) extends his earlier model both conceptually by adding a level of processing as well as by increasing the number of systems that comprise each level of processing. So, instead of a single Autonomous Mind, Stanovich (2009, p. 56) proposes “a *set* of systems in the brain that operate autonomously in response to their own triggering stimuli and are not under the control of the analytic processing system [i.e., System 2]”. This heterogeneous set, to which he refers as The Autonomous Set of Systems (TASSs), contains systems that are related in terms of their style of functioning (i.e., automaticity) rather than related by modularity. The proposed tri-process CNDS model incorporates two systems of Automatic Mind: the state-impulsive system comprising the state impulsivities and the trait-impulsive system comprising sensation-seeking and reward/reinforcement sensitivity (See also Kahneman, 2003, 2011; Shea et al., 2014).

Type 2 processing is divisible into two sorts of operation, each characteristic of a “kind of mind” (Dennett, 1996). The *Algorithmic Mind* involves individual differences in fluid intelligence, that which is measured by IQ tests, while the *Reflective Mind* involves individual differences in rational thinking dispositions. Rationality is broader than intelligence, requiring well-formulated desires (goals), highly calibrated beliefs and the ability to act on them in order to achieve the goals. It is, therefore, closely associated with the elements that Bickel et al. (2012b) position as components of the Executive system which, in their analysis, found no corresponding antipodal response in the Impulsive system.

The distinction between the two Type 2 systems posited by the tri-process theory rests on several functional differences. The key function of the Reflective Mind is the inauguration of the call to begin cognitive simulation or hypothetical reasoning. The key operation of the Algorithmic Mind in this is the decoupling it carries out. Decoupling is cognitively demanding, assisted by language which provides “the discrete representational medium that greatly enables hypotheticality to flourish as a culturally acquired mode of thought” (Stanovich, 2009, p. 63). Hypothetical thought requires the representation of assumptions for instance and linguistic forms like conditionals readily allow this. Decoupling abilities differ in their recursiveness and complexity. Decoupling makes it possible to distance oneself from the representations so they can be reflected on and improved. Decoupling is therefore the key function of the algorithmic mind. It is clearly a System 2 operation in that its operation occurs serially and incurs high computational expense. The literature on executive function and working memory, he argues, supports the view that the main function of Algorithmic Mind is to achieve decoupling among representations while conducting cognitive simulation.

The cognitive control exerted by elements of the tri-processual model is justified as follows. System 2, representing analytic mind, contains two levels of functioning: the *algorithmic* level and the *reflective* level. TASS systems will function on a short range basis unless this is overridden by the Algorithmic System which give precedence to the long range goals of the analytical system. These latter reflect the goals of the person and the “epistemic thinking dispositions”. But these goals and dispositions must arise at a level superior to that of the Algorithmic System namely in the Reflective System “a level containing control states that regulate behavior at a high level of generality” (Stanovich, 2009, p. 57). This distinction of analytical systems gives rise to a tripartite system of cognitive processing. The Algorithmic Mind and the Reflective Mind share properties (such as capacity-limited serial processing) that distinguish them from the Automatic Mind (Stanovich, 2009, p. 58) But Algorithmic Mind and Reflective Mind can still be distinguished from one another, especially if we think in terms in relation to the impulsive executive systems. If these two systems of Automatic Mind are in conflict or competition, as the CNDS model proposes them often to be, any adjudication between them that results in a compromise or balanced influence on behavior will have to be done at a superordinate level of processing. It must draw on system goals and strategic procedures that are not the property of either of these systems but of a level of processing that is superior to both of them. This is the Reflective Mind.

Stanovich (2009) argues that measures of the executive functions actually draw upon elements of the Algorithmic Mind rather than the Reflective Mind. While the term “executive” seems superficially to suggest that these functions concern the highest level of mind, Reflective Mind, the tasks used by cognitive scientists to assess executive function actually test skills that result from Algorithmic Mind. Research in cognitive psychology in particular has been concerned with tasks that involve algorithmic level decoupling abilities: stanovich mentions “stop signal paradigms, working memory paradigms, time sharing paradigms, inhibition paradigms” which are highly suggestive of the components of executive function that Bickel et al. (2012b) found to be antipodal to state impulsivities. Individual differences in Reflective Mind capabilities are scarcely involved in these tasks if at all. The Reflective Mind, especially with respect to its involvement in epistemic regulation and cognitive allocation is involved in cognitive control at a level beyond that of the computational capacity to maintain decoupling. Stanovich (2009, p. 66) argues, therefore, that the executive functions have been misnamed: they are essentially *supervisory processes*, he maintains, based on eternally provided rules rather than internally inaugurated decision-making. By contrast, Reflective Mind is involved in setting “the goal agenda” or in operating at the level of epistemic regulation which he defines as “directing the sequence of information pickup”. Executive functions are not engaged in this kind of work.

While the executive system belongs to the Algorithmic Mind, however, it does not constitute the Algorithmic Mind exclusively. The executive system is fundamentally involved in the overriding of the Automatic Mind but other functions of Algorithmic Mind such as the execution of decoupling are not carried out by the executive system. Similarly, the impulsive system is not the sole element of TASS; the trait impulsivities (sensation seeking and reinforcement sensitivity) also belong to TASS and are involved in moderating the tendency toward impulsivity or self control at the behavioral level. Hence, even the Type 1/Type 2 dichotomy recognizes a complexity that goes beyond that of the original CNDS model. However, Stanovich (2009, 2011; Stanovich et al., 2012) argues for a further distinction, this time between the kinds of processing for which Type 2 systems are severally responsible, which if accepted complicates the division between impulsive and executive systems made by the CNDS model. The interaction of Type 1 and Type 2 processing is evinced by the capacity of the second to prevent the automatic responses inherent in Type 1 processing to engender impulsive behaviors that result in suboptimal outcomes. “Better” responses depend on Type 2 *hypothetical reasoning* in which the individual builds models of the world and performs cognitive simulations on them. Stanovich et al. (2012, p. 787) comment, “When we reason hypothetically, we create temporary models of the world and test out actions (or alternate causes) in that simulated world,” words reminiscent of Popper’s observation that “our conjectures, our theories, die in our stead!” (Popper, 1977) In order to effect this cognitive functioning, Type 2 processes can override those of Type 1, interrupting and suppressing Type 1 functioning and then substituting alternative responses. Moreover, in order to form simulations, it is necessary to *decouple* simulated models from the real world so that they can be manipulated independently. This initiation of decoupling secondary representations from the world and maintaining them while simulation occurs is a Type 2 operation.

Having “taken TASS oﬄine,” the Algorithmic Mind initiates decoupling which enables cognitive simulation to take place. The outcomes of this are reviewed by Reflective Mind which initiates change in serial associative cognition which influences Algorithmic Mind to develop a response. The initiation of serial associative cognition illustrates that while all hypothetical thinking involves analytical mind, not all the actions of analytic mind involve hypothetical thinking. Serial associative cognition is somewhat shallow thinking, “cognition that is not rapid and parallel such as TASS processes, but is nonetheless inflexibly locked into an associative mode that takes as its starting point a model of the world that is *given* to the subject” (Stanovich, 2009, p. 68, 70). Serial associative cognition “is serial and analytic … in style, but it relies on a single focal model that triggers all subsequent thought.” Hypothetical thinking constitutes a vital *reasoning* function. The reflective and algorithmic processes of the analytic mind each have a key function within this process. Hypothetical thinking is closely related to the notion of TASS override. The analytic system must take TASS-initiated tendencies toward behavior oﬄine and replace them with a more appropriate response. Such better responses come from cognitive simulation where they can be tested; only if they survive that will they be adopted.

### Triple Processing in the Context of CNDS

**FIGURE 5:**
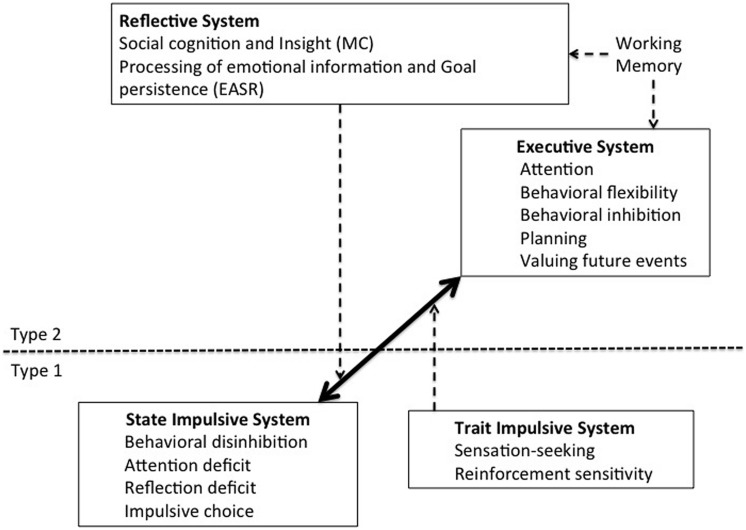
**Metacognitive Control of CNDS.** This model depicts two Type 1 impulsive systems which are responsible, respectively, for the state impulsivities that are antipodal to the elements of the Executive System, and the trait impulsivities that modify the relationship between the State Impulsive System and the Executive System. This key relationship is shown by the bold arrow. The Executive System exerts Type 2 influence on this relationship which is modified also by the action of the Type 2 Rational System which encourages co-operation between the State Impulsive System and the Executive System. The Type 2 systems draw upon Working Memory for their operations. The relationship between the State Impulsive System and the Executive System (bold arrow) is the immediate precursor of the degree of temporal discounting exhibited in the individual’s behavior.

It is feasible, therefore, to develop the CNDS model by incorporating MC and EASR as components of a level of processing superordinate to those of the impulsive and executive systems (**Figure 4**). This figure depicts two Type 1 impulsive or TASS systems: the first comprises the state impulsivities that Bickel et al. (2012b) showed to be antipodal to components of the Executive system; the second is composed of the two trait impulsivities, sensation seeking and reward sensitivity, that are not linked antipodally to elements of the Executive system. They are shown here as exerting modifying influences on the relationship between the State Impulsive System and the Executive System. This key relationship is shown by the bold arrow. The Executive System exerts Type 2 influence on this relationship which is modified also by the action of the Type 2 Reflective System which promotes balance between the State Impulsive System and the Executive System. The Type 2 systems draw upon Working Memory, another element ascribed to the Executive system in the original CNDS model (**Figure 1**) which has no antipodal complement in the Impulsive system, for their operations. For this reason, it is shown separately from the Type 1 and Type 2 systems in **Figure 5.** The relationship between the State Impulsive System and the Executive System (bold arrow) is the immediate precursor of the degree of temporal discounting exhibited in the individual’s behavior.

Individual differences in sensation seeking and reward or reinforcement sensitivity, which may derive from the individual’s neurophysiology and/or learning history, are posited as moderating the relationship between the impulsive and executive systems. Sensation seeking is understood by Zuckerman (1979, 1994) as a preference for sensations and experiences that embody variation, novelty, and complexity, together with a willingness to incur physical and social risks in order to gain such experience. Reinforcement sensitivity reflects individual differences in susceptibility to reinforcing and aversive stimuli. Reinforcement sensitivity theory (RST; Corr, 2008; Smillie, 2008) relates propensity to behavior not only to the stimuli that have been consequential on such behavior in the past but also to the mediating neurophysiological events that are the immediate precursors of responding.

Left to itself, the Automatic Mind will act via the state impulsivities, in the absence of any influence of the behavioral inhibition, planning, and attention-maintaining tendencies of the Algorithmic Mind: the result will be a failure to reflect on the longer-range outcomes of immediate behavior, so that the resulting behavior reflects a preference for SSR over LLR. Pursuit of this short-range interest can be overcome only by an intervention of the Reflective Mind which initiates override of the Automatic Mind via the Algorithmic Mind. Acting in response to the Reflective Mind’s initiation of override, the Algorithmic Mind activates its executive functions that counter impulsivity (paying attention, drawing on behavioral flexibility and disinhibition, planning, and valuing future events) and which enable longer-term interests to be explored and pursued. Override, which thus consists in the countering of the immediate short range of the Automatic Mind by exercise of the executive functions, does not of itself result in the formulation of a plan for longer-term behavior, however. Planning with foresight entails that the Reflective Mind also initiate the decoupling of the representations for which the Automatic Mind and Algorithmic Mind are responsible so that simulation of alternative courses of action can take place. Simulation makes possible the hypothetic thinking that permits these alternatives to be generated and tested: an apparently satisfactory plan (one that is strategically and consistent with long-term goals and capabilities) engenders a response from Reflective Mind such as the pursuit of a longer-term objective in place of the impulsive action which unencumbered Automatic Mind would have produced.

The trait impulsivities can promote or impede the operations of either the Automatic Mind or the Reflective Mind, working toward the generation of either the short- or long-range interest. Trait impulsivities, sensation-seeking and reward sensitivity, are based on individual differences which are susceptible to learning history as well as the neurophysiological basis of behavior. How the trait impulsivity system works is debatable but it may be responsible for the *style* of thinking characteristic of or preferred by an individual, his/her tendency toward an analytical or intuitive approach to problem solving (Sadler-Smith, 2009). This would set limits to an individual’s range of actual behaviors. Imagine a hypothetical range of behaviors from the most impulsive to the most executively controlled which contains all the actual ranges of behavior of which individuals in the population are capable. The actual range of any individual will be a subset of this. The extent of the actual subset that is the behavioral range of any individual will reflect his/her cognitive style especially as it is determined by sensation-seeking and reinforcement sensitivity, the propensity of his/her behavior to be reinforced by highly arousing stimuli and immediate reward.

The tri-process configuration captures well the requirements of the CNDS model, especially in portraying those of its elements that have been shown to be antipodal, those that remain after the establishment of antipodality has been exhausted, and the relationships among them. The tri-process model comprises an Automatic Mind which responds rapidly to environmental circumstances (which captures well the imperatives of the impulsive system posited by the CNDS model). This Automatic Mind can, however, be checked by the Algorithmic Mind (that includes the executive system which has precisely the antithetical imperatives required to counter the impulsive tendencies of Automatic Mind). The Algorithmic Mind’s countering the tendencies of the Automatic Mind relies in turn on its being directed by the Reflective Mind to override the Automatic Mind in order to inaugurated the decoupling of representations based on reality so that the procedure of simulation via hypothetical thought can occur. In simulation, alternative behaviors that might be enacted can be examined in terms of their outcomes in the short and long term. The information so gained is fed back to the Reflective Mind which inaugurates action. The Reflective Mind has additional functions which include monitoring environmental circumstances and being aware of the likely response of the Automatic Mind to them in order to initiated decoupling and simulation. These are not the functions of the Algorithmic Mind of the tri-process theory or the executive system of the CNDS model.

### A tri-Process Framework

The CNDS model portrays normal and addictive behaviors as the outcomes of interaction between an impulsive system based on limbic and paralimbic brain regions and an executive system based in PFC. The interaction is indexed behaviorally by the steepness of the temporal discounting an individual’s decision-making exhibits. Several of the elements of the impulsive and executive systems in the model are antipodal in terms of their definition, measurement, application to populations of addicts, and neurophysiological substrates. Specifically, the state impulsivities of the impulsive system and the elements of the executive system responsible for the CTOB display antipodality. Configuring the remaining elements of the model according to developments in multi-process theories of cognition does not detract from the CNDS model but extends its capacity to explain normal and addictive behaviors. It has, therefore, been argued that elements of the impulsive and executive systems that do not correspond in this way, constitute additional systems that provide a more comprehensive understanding of the ways in which the interaction of the competing systems eventuate in behavioral responses. In the tri-systems theory of Stanovich (2009), a third level of processing (Reflective Mind) provides a mechanism through which the conflicting imperatives toward impulsivity and restraint of more basic systems can be managed and superseded. *Metacognition* (MC) and EASR, elements of the CNDS model’s executive system which find no antipodal correspondents in the impulsive system, contribute to this third level. The state-impulsive system belongs to what Stanovich terms Automatic Mind, while the executive system belongs to Stanovich’s Algorithmic Mind. The trait impulsivities, *sensation-seeking* and *reward sensitivity*, which have no antipodal correspondents in the executive system form an additional system within Automatic Mind. This trait-impulsivity system moderates the individual’s behavioral output which manifests in a rate of temporal discounting. This move receives support from Stanovich and West’s (2003) argument that there are individual differences in how effective Algorithmic Mind is in overriding Automatic Mind. The removal of the trait impulsivities from the impulsive system to form another TASS makes these variables’ influence more coherent; if they act negatively on the relationship between the impulsive and executive systems, they can undermine overriding, decoupling and perhaps simulation. Ross et al. (2008) note that some individuals may simply be incapable of bundling. One of the causes of this deficiency may be the overvaluation of reinforcers that arises from a tendency toward seeking unusually high levels of arousal and the particularly strong sensitivity to reward.

### A Proposal for Empirical Research

The composite model summarized in **Figure 5** proposes that the outcome of conflict between the State Impulsivity System and the Executive System is the immediate precursor and cause of the rate of temporal discounting exhibited in the individual’s behavior. The model suggests further that the Reflective System is responsible for the extent to which social insight and emotional control exert an inhibiting influence on the tendency toward impulsive behavior (choice of SSR over LLR); it suggests also that the Trait Impulsivity System is responsible for the degree to which the individual is inclined to control his/her impulsivity and that sensation seeking and reinforcement sensitivity are especially potent in this regard. The variables that compose the Reflective System and the Trait Impulsivity System reflect individual differences in self-control versus impulsivity. The precise measures of the effects of the Reflective System and Trait Impulsivity System remain to be empirically determined. The task of empirical research inspired by the model is to determine how and to what extent these variables impact the rate at which temporal discounting occurs and therefore the degree of balance the individual exhibits between the operation of the State Impulsive System and the Executive system. The following suggestion for empirical research is indicative in general terms of the feasibility of a research program that would facilitate the critical examination of hypotheses drawn from the model. Its principal objective at this stage is to demonstrate that the model is amenable to empirical investigation and is falsifiable in principle.

Stanovich argues that Reflective Mind involves the exercise of a cognitive style that influences the overarching approach an individual assumes in the pursuit of problem solving and decision making. As I have discussed elsewhere (Foxall, 2014b, 2016), one approach to the empirical delineation of cognitive style is provided by Kirton’s (2003) adaption-innovation theory and measure. On this theory, extreme adaptors pursue solutions to problems within tried and tested frameworks of experience-based analysis and conceptualization and are likely to discount the future less steeply than innovators who seek solutions in novel and outlandish proposals which entail steeper discounting. The adaptor is likely therefore to exhibit greater capacities for social cognition and insight, to process emotional information in a more constrained fashion, and to persist in the pursuit of a goal once it has been adopted. The innovator is more likely to rely more on his/her own notions of how pursuit of a specific behavior would generate effective consequences, to be more emotionally involved in the advocacy of his/her ideas, and to be more easily deflected from current goals in favor of novel objectives. The behavior patterns typical of adaptors and innovators may also be grounded in separate neurophysiological regions (van der Molen, 1994). There is therefore scope for empirical research which seeks to test the hypotheses (1) that adaptors exhibit a lower rate of temporal discounting on specified decision tasks than will innovators, and (2) that these cognitive styles are associated with the innervation of distinct neurophysiological regions that reflect the brain bases of high and low levels of temporal discounting.

Similar investigation is feasible by means of psychometric measures of sensation seeking and reinforcement sensitivity which may be employed to monitor the trait impulsivity of individuals engaged on tasks involving decisions that reflect differing rates of temporal discounting. Higher levels of both of these traits would be expected to associate with steeper temporal discounting and also to be linked to distinct brain regions. To the extent that sensation seeking and reinforcement sensitivity are captured by the adaption-innovation spectrum, Kirton’s measure of cognitive style may also suffice for the investigation of these dimensions of trait impulsivity. The ultimate aim of research of this kind is to establish double dissociations (a) between components of cognitive functioning and rate of temporal discounting, and (b) between cognitive functions and neurophysiological activation. Initial investigation (Foxall and Yani-de-Soriano, 2011) suggests that the thorough empirical examination of the model would require psychometric investigation of the individual traits that comprise the Reflective System and the Trait Impulsive System in order to present a more fine-grained analysis of the relationships proposed by the model. Situational variables, notably the specific nature of the decision under investigation would likely influence the extent to which consumers discount the future in addition to the contribution of their fundamental cognitive styles.

## Discussion

### Bundling in tri-Processual Perspective

Of the three components of the CNDS model – neurophysiology, decision-making (cognition), and behavior – cognition probably has received least attention. The foregoing discussion supports the conclusion, however, that if the elements of the CNDS model are configured in accordance with multi-process theories of cognitive control such as that of Stanovich (2009) the cognitive implications of the model can be made explicit. This proposition can be tested by applying the framework presented in **Figure 5** to explicate the idea of bundling.

Bundling involves an individual’s adoption of a rule in order to overcome the tendency to select the inferior of two rewards as a result of discounting the future hyperbolically. The rule prescribes that one consider all of one’s choices between pairs of reward of this kind in a way that makes one’s present choice the precedent for later choices. In this way the individual precommits him/herself to act in a particular manner by recognizing that selecting an entire series of LL alternatives motivates him/her to avoid temporary preferences for SS options when they arise (Ainslie, 1992, 2001, 2007; Ainslie and Monterosso, 2003).

An individual who considers each choice between an SSR and a LLR as it arises is likely as we have seen to initially prefer the latter but to switch preferences when the value of the former is magnified by the fact of its imminent availability. This pattern is recursive: good resolution is followed by akrasia, not once but repeatedly. If this person resolves to consider the sum of all future SSRs in relation to the sum of all future LLRs, the conclusion is that future LLRs will cumulatively outstrip future SSRs. Crucially, the sum of the series of LLRs is also greater than the first SSR that will be encountered in the series, i.e., when it becomes immediately available, a comparison which makes acceptance of the LLR on this first occasion easier. Insofar as this first choice is predictive of later choices, following this rule makes a series of LLR choices more probable. The bundling strategy is not only theoretically defensible but also practically efficacious (Ross et al., 2008).

Both the Automatic Mind (embodying the impulsive system) and the Algorithmic Mind (executive system) are involved in this process. However, bundling requires in addition an array of mental operations which can be most appropriately understood by reference to the tri-process model we have considered. These operations include (i) holding the immediately available behavioral option in mind, (ii) holding the array of long range behaviors and their outcomes in mind, (iii) summing the outcomes of the long range behaviors, (iv) bringing the summed outcomes into comparison with that of the short range outcome, and (v) adjudicating between them. These operations cannot be carried out within either the impulsive system or the executive system. Neither has the capacity to undertake these tasks. Moreover, since the short-term and long-term interests depicted in terms of temporal discounting by picoeconomics, exist by definition at different times, the only way in which they can be brought together is mentally, specifically through the medium of imaginative or hypothetical thinking. Representations of the two interests must be created and allowed to impinge on one another. Hence, the process of bundling is that described by Stanovich as requiring the decoupling of the Automatic Mind and the simulation by means of hypothetical thinking of alternative scenarios for future behavior. These operations require the monitoring of the behavioral tendencies of Automatic Mind in light of environmental contingencies (which must also be monitored beyond the level of the impulsive system), the initiation of override of the Automatic Mind, and the initiation of simulation via decoupling in which alternatives to the immediate uncritical pursuit of short-term gratification are hypothesized and evaluated. The only area of mind that can initiate these procedures is the Reflective Mind. The Algorithmic Mind cannot undertake such monitoring and initiating. Its functions are regulatory and supervisory rather than innovative, and bundling depends on hypothetical thinking that brings a multiplicity of long range outcomes and short range outcomes into the same arena and allows them to impinge on one another so that a calculation based on the valuation of the separate outcomes and a selection the appropriate action can be made.

There is another reason why the tri-process model is particularly relevant to the analysis of normal and addictive decision-making by means of the CNDS model and picoeconomics. Neither the state-impulsive system element of the Automatic Mind nor the executive system of the Algorithmic Mind can adjudicate between the imperatives of immediate gratification that fulfill the short-range interest embodied in the former and the delayed benefits that fulfill those of the long-range interest. Both the CNDS model and picoeconomics are enhanced by the inclusion of Reflective Mind in the overall system of decision-making they posit. The Reflective Mind is a kind of *present self* that can hypothesize about the behaviors of one’s *past self* and *future self.* Hypothetical reasoning requires that representations of the real world not interfere with representations of imaginary situations. In comparing the pleasure to be obtained by ingesting a recently acquired drug with the deleterious consequences of a series of binges in the future, it is necessary to differentiate clearly the monetary cost of the newly obtained supply of the drug from the imagined emotional and social as well as financial costs of sustained consumption that would be the outcome of binging. These abstract operations require the participation of a Reflective Mind.

### Multiple Selves or Incompatible Interests?

This account of bundling operations implies that the rational decision-making element of the model shown in **Figure 5** is Reflective Mind. the impulsive and executive systems (inherent in Automatic Mind and Algorithmic Mind, respectively) appear to be largely neurophysiological systems that are under the ultimate control of Reflective Mind. It is here that personhood or agency is located: while it may experience the conflict of having to decide between alternative interests by determining the content of the utility function that will be the outcome of its behavior, it is a single person.

Dual process models such as CNDS contain the conflict between short- and long-range interests within warring impulsive and executive systems; picoeconomics, whilst open to multiple selves, also tends in practice largely to confine its deliberations to these two categories of mental operation. But there are elements of the executive system, as defined in **Figure 2**, that tri-process theories such as that of Stanovich (2009) suggest play an overarching role in the relationships between the systems it characterizes as Automatic Mind and Algorithmic Mind and which, respectively, embrace the impulsive and executive systems. The restructuring of the model components, achieved in **Figure 3**, proposes that MC and EASR would constitute part of this higher level system, the Reflective Mind, which would be involved in the regulation of the Automatic Mind which otherwise would respond to environmental stimuli spontaneously or impulsively. The regulation imposed by Reflective Mind would take the form of its “innervating” Algorithmic Mind to initiate the overriding of Automatic Mind and the decoupling of mundane mental representations so that the simulation of hypothetical futures can be accomplished. Reflective Mind is also portrayed in Stanovich’s theory as receiving the outputs of simulation and effecting a response at the level of the entire organism.

Caution is essential on the part of psychologists, whether cognitive or behavior analytical, in their treatment of models of this kind. Models that depict cognitive operations necessarily deal in unobservables and there is a danger that these will be multiplied without a firm empirical basis being provided for them. In applied areas such as the treatment of addiction and other forms of excessive consumption it may be necessary, however, to use ascriptions of thought processes to individuals in order to understand fully their behavior possibly as a prelude to predicting and/or modifying it. There is, moreover, little in the modeling which has been the subject of this paper that could not be captured, though perhaps less economically, in the language of behavior analysis and in particular that of verbal behavior and rule-governance. There need be no internecine conflict between adherents of different vocabularies as long as the analysis is comprehensible to all in terms of their several theoretical viewpoints. This is especially the case if the new analysis proves useful at the level of effecting the prediction and control of behavior but it is also justified if its principal contribution is the furtherance of understanding of the processes involved in shaping and maintaining that behavior.

Where does this model-building lead in terms of resolving the question of multiple selfhood that opened this paper? Ross (2009; see also Ross, 2005) argues that there are present at t_1_ and t_2_, respectively, different *agents* as is demonstrated by there being different utility functions at each time. However, useful this agential distinction process is as an analytical device, the degree of difference between *selves* or *persons* must not be exaggerated. To argue that different persons or selves exist at t_1_ and t_2_ may be self-defeating since it is only by establishing a considerable degree of continuity between the person who exists at t_1_ and the person who exists at t_2_ that we can comprehend why intrapersonal conflict arises. If the t_1_ and t_2_ persons or selves are remote from one another we can argue that neither is bound by what the other has done (Hanson, 2009). If the selves can establish this degree of moral separateness, it is difficult to see how the motivational conflict engendered by the contemporaneous existence of competing interests necessary for picoeconomics can come about.

What exists at t_1_ and t_2_ is the organism; without the assumption of at least this degree of continuity there would be no conflict. The question is whether this self that persists is the agent or whether “interests” or “selves” existing within the person can be thought of as agents each of which has a separate utility function. I would argue that the person who exists at t_1_ and t_2_ has different interests or motivations on each occasion because he/she is facing different contingencies of reinforcement and punishment. It is unnecessarily metaphysical to argue that two persons or agents are involved: it is one person or agent with conflicting interests. The interests have neurophysiological correlates within the person which form a central aspect of the impulsive and executive systems; these neurophysiological events are to be understood at the sub-personal level of exposition. The cognitive dimensions of the Automatic Mind and Algorithmic Mind which include these and other systems are to be understood, however, at the personal level of beliefs, desires, perceptions, and emotions. Not only, contra Dennett (1969, 1987), can the intentionality that properly belongs at the personal level not be ascribed to sub-personal entities (Foxall, 2007); it is also not possible for the interests to have utility functions of their own and thus be considered agential. Rather, a person’s interests determine his/her preference structure (as revealed in his/her choice *behavior*) which eventuates in his/her utility function (the final configuration of the consequences of his/her behavior (Rachlin, 1994, 1989). What changes from t_1_ to t_2_ is the contingencies of reinforcement with which the individual is faced; these have the effect of modifying his/her utility function. The preferences embodied in his/her behavior reflect the dominance of either the impulsive and executive system (or the Automatic Mind and Algorithmic Mind of which they are subsets) at that time. The Reflective Mind is the personal forum within which the deliberations regarding the alternative behaviors available and their likely outcomes takes place. It is within the person that conflict occurs and is felt.

The rational individual for whom akrasia is a commonplace experience is not, therefore, two persons, or agents in the course a day, but a single person who encounters differing situations and changes his preferences accordingly; these preferences are revealed in the earlier verbal behavior which values highly constructive work and in the subsequent physical behavior which values recreation. These contingencies of reinforcement are each advantageous in their way at different times and insofar as he/she is conscious of them they signal the benefits that will derive from his/her behavior. These benefits form my interests. There is no need to translate these extrapersonal interests into intrapersonal homunculi that compete. What compete are (i) the differing contingencies of reinforcement (at the super-personal level), and (b) the differing neurophysiological tendencies (at the sub-personal level). The personal level is concerned with acting upon one or other of these, and/or adjudicating between them. In the course of debating different courses of action, any rational person may experience cognitive discomfort, feel as though they are being wrenched first by one alternative then the other as they participate in “making a choice.” But at no time is this individual anything other than a person facing incompatible options who finds this situation aversive. He/she has only one utility function at a time, by virtue of being able to perform only one behavior at a time and his/her utility function is the outcome of that behavior.

## Conclusion

This paper has argued that the CNDS model’s obvious strengths can be enhanced through consideration of the categorial structure of the model and the functions of its components. Hence, the formulation shown in **Figure 5** seems more closely aligned with the results of the investigation of the antipodality of the impulsive and executive systems undertaken by Bickel et al. (2012b). The question that arises from that exercise is where the components of the impulsive and executive systems (**Figure 2**) that have no antipodal correspondents should be positioned within the CNDS model. The proposal to remove MC and EASR from the Executive System and accord it a superordinate role in cognitive control of (a) the impulsive and executive systems and (b) overt behavior suggested in **Figure 3** is borne out by the theoretical reasoning advanced by multi-process modelers such as Stanovich (2009). Recent theoretical development suggests also that several systems comprise Automatic Mind (Stanovich, 2011) and this offers a role to the state impulsivities that do not exhibit antipodality with any of the elements of the Executive System depicted in **Figure 2.** A final tri-process model which incorporates the restructured components of the CNDS hypothesis is put forward in **Figure 5.** Although this model does not essentially contain any components not already present in the original CNDS model (**Figure 2**), it aims to present their interrelationships in a way that is consistent with their functions in controlling the imperatives of impulsivity and self-control and the individual’s behavior.

Three themes emerge from this analysis. The first is the implications for the CNDS model of considering cognitive control of behavior in light of the tri-process theory. The second is the capacity of the tri-process depiction of neuro-behavioral decision-making to enhance understanding of addictive behavior and its resolution. This is discussed by Foxall (2016) in terms of the mental operations that are presupposed by picoeconomic bundling and their incorporation in the model presented in **Figure 5**, as is the nature of the multiple agents apparently involved in the breakdown of will and its resolution is discussed in the context of the model.

## Author Contribution

GF is entirely responsible for the paper.

## Conflict of Interest Statement

The author declares that the research was conducted in the absence of any commercial or financial relationships that could be construed as a potential conflict of interest.

## Footnotes

1 Behavior analysts have conducted a large volume of research on temporal discounting and the matching phenomena which underlie it (Ferster and Skinner, 1957; Herrnstein, 1997). For reviews of this research, see Madden and Bickel (2010), Dallery and Soto (2012), Grace and Hucks (2012), Hursh et al. (2012), Jacobs et al. (2012), Foxall (2016). See also Foxall (2015) and the special issue on Operant Behavioral Economics this introduces.

2 Recent research indicates a far more complicated picture than this. LTP is currently understood as no more than a possible molecular mechanism of learning (see, for instance, Migaud et al., 1998; Uetani et al., 2000). The role of LTD, which has been correlated with learning, is of particular importance. Present knowledge on synaptic plasticity and learning performance, incorporating the learning of drugs’ capacity to reward, indicates that LTP and LTD tend toward an optimal balance which may influences memory performance. Memory formation may also be subserved by hippocampal network oscillations in the theta, gamma and high-frequency range and the activity of place-, grid-, time-, and reward-related cells in the brain (Pan et al., 2005; Eichenbaum, 2014; Moser et al., 2015; Sanders et al., 2015).
